# Etiology and Treatment of Acne Vulgaris

**Published:** 1902-12

**Authors:** F. H. Beadles

**Affiliations:** Richmond, Va., Professor of Botany and Pharmacognosy and Lecturer on Diseases of the Skin, Medical College of Virginia


### ETIOLOGY AND TREATMENT OF ACNE VULGARIS.*

BY F. H. BEADLES, M.D., RICHMOND, VA.,

Professor of Botany and Pharmacognosy and Lecturer on Diseases of the Skin, Medical College of Virginia.

This paper has been written to review in the simplest manner our knowledge of the etiology and treatment of this apparently simple yet distressing affection of the skin. I think I use the word "distressing" advisedly, for this disease robs many an individual of that coveted possession—physical beauty.

The most important etiological factors in the production of acne vulgaris are : age, gastro-intestinal disturbances, disturbances of the generative organs.

It is common to describe the changes incidental to puberty in both sexes as a frequent cause of this condition, but a physiological crisis is rarely a disease factor, unless the full and normal development of the period be prevented by accident, disease or malnutrition, or by excessive demands upon the vital organs. There is no doubt that the increased growth of hair in both sexes at puberty, occasions an unusual activity of the sebaceous glands. While thousands pass the age of puberty without being affected with acne, the disease, none the less, is prone to appear first at this time of life, and, if not properly treated, to spontaneously disappear when full maturity of the body is reached.

That gastro-intestinal disturbances play an important part in the production of acne is demonstrated by the frequent existence of an acne in individuals affected with indigestion, constipation and dyspepsia, or those who indulge excessively in cheese, pastry, sweets or highly seasoned foods.

There is no doubt that a close physiological connection exists

*Read before the Richmond Academy of Medicine and Surgery, October 28, 1902.

between the genital organs, their functions, and the skin, not only in man, but also in the lower animals. It is common to note the aggravation of an acne just before or during the menstrual flow. Masturbation and continence have each been blamed as excitants of this condition. While the former of these itself does not cause acne, its well-known effects on the nervous, moral and physical condition of growing youths would sufficiently account for any part it may have in producing this affection. Neither is there proof that continence is a cause of acne. Therefore, it is safer for us to attribute their presence to bad sexual hygiene rather than to masturbation or continence.

Treatment: It is an acknowledged fact that acne is a remedial disease when properly managed. It is true that scars of ancient ravages are sometimes almost indelible, but even these may be smoothed down by continued treatment so that yearly they become less conspicuous and disfiguring.

The constitutional treatment depends for success upon the discovery of the cause of the disease. In a large per cent. of cases the question of diet is a most important one. A marked improvment generally follows the reduction of the amount of food ingested, particularly meats; while a diet restricted to milk, bread, fish, fruits and the lighter vegetables, will usually benefit the most obstinate cases.

An essential part of the treatment is the daily sponging (except during the menstrual epoch) of the entire surface of the body except the face (which requires special treatment) with water as cool. as can be tolerated, followed by brisk rubbing with a coarse towel until the skin is glowing.

Of the agents used for their specific action on the skin the most commonly used is arsenic. It is a drug which is known to exert an influence upon the epithelia of the skin; and upon these, so far as its therapeutic effect is concerned, only when they are the seat of subacute and chronic exudations. By comparison of the experience of experts, it has been shown that the common practice of giving arsenic for this condition is both harmful and irrational, not only because of its effect in producing cutaneous congestion, but also on account of the reliance placed in it to the exclusion of other and better methods of treatment.

Calcium sulphide, also long esteemed, is now classed with the ludicrous specimens of therapeutic empiricisms.

I shall not attempt to review the various plans of local treatment which have been advocated for this condition, but rather to outline a single plan, which, when followed closely, is productive of the happiest results.

In determining the method of treatment, it is well to consider three conditions which are usually present in the skin of such patients—hyperkeratosis, flaccidity of the muscles and hypersecretion.

As the face is the commonest seat of the disease, for the purpose of description it may be considered as the part affected. First, the surface is rendered aseptic. A massering ball is then rotated freely over the surface, deep pressure being made over the affected region with the result of bringing into view groups of previously inconspicuous comedones, which are in turn, removed with an extractor. A ring curette is next drawn over the lesions, expressing their contents and stimulating others to activity. The subsequent bleeding is encouraged by sponging with hot water. The patient is then directed to bathe the face thoroughly for ten minutes, just before retiring, with spirits of green soap and water as hot as can be borne; after which the face is wiped dry and some astringent lotion, which is allowed to evaporate on the surface, is applied. A lotion which I have found highly beneficial, is composed of the following:

| | | |
|---|---|---|
| ℞ | Zinci sulphas | drachms j. |
| | Potass. sulphuret | drachms j. |
| | Aquæ rosæ, q. s. | ounces iv. |
| M. | | |

